# Significant wave height prediction from X-band marine radar images using deep learning with 3D convolutions

**DOI:** 10.1371/journal.pone.0292884

**Published:** 2023-10-30

**Authors:** Ji-Woo Kwon, Won-Du Chang, Young Jun Yang

**Affiliations:** 1 Department of Artificial Intelligence Convergence, Pukyong National University, Busan, Republic of Korea; 2 Department of Marine Mobility, Tongmyong University, Busan, Republic of Korea; Sunway University, MALAYSIA

## Abstract

This research introduces a deep learning method for ocean wave height estimation utilizing a Convolutional Neural Network (CNN) based on the VGGNet. The model is trained on a dataset comprising buoy wave heights and radar images, both critical for marine engineering. The dataset features X-band radar images sourced from Sokcho, Republic of Korea, spanning from June 1, 2021, to August 13, 2021. This collection amounts to 72,180 three-dimensional images, gathered at intervals of approximately 1.43 seconds. The data collected was highly unbalanced in terms of wave heights, with images of lower wave heights being more common. To deal with data imbalances in the wave height datasets, we categorized the data into three groups based on wave heights and applied stratified random sampling at each level. This approach balances the data patches for each training iteration, reducing the risk of overfitting and promoting learning from diverse data. We also implemented a system to protect data in groups with fewer instances, ensuring fair representation across all categories. This study presents a deep learning regression model for predicting wave height values from radar images. The model extracts features from sequences of 64 radar images using three-dimensional convolutions for both temporal and spatial learning. Using three-dimensional convolutions, the model captures temporal features in radar image sequences and provides accurate wave height estimates with an RMSE of 0.3576 m. The study derived results using radar images under different wave height conditions for 74 days to ensure reliability.

## Introduction

Ocean wave information is important in a wide range of areas that are directly or indirectly affected by the sea, including safe ship navigation, various tasks on both offshore and onshore platforms, fishing, and marine sports. This information is largely composed of the height, period, and direction of the waves. In engineering, the significant wave height or peak wave height is used to secure statistical significance. The wave height information is obtained by integrating the energy value in the wave spectrum. Fourier transform has been used for a long time to calculate wave height information, which is used to identify periodic characteristics of information measured using buoys and radar [1].

While buoys are the most widely used tool for measuring wave height and achieving highly accurate results, they require mooring and are fixed at a single point for measurement purposes. Therefore, they cannot be used for moving ships, and high installation and operation costs are incurred in deep water areas. With recent advances in electronic communication technology, wave measurements based on remote measurement technology, such as radar are becoming more common [2]. Wave measurement technology has been developed based on an analysis of X-band radar images from satellite X-band synthetic aperture radar (SAR) and an X-band slot array antenna for ships [3].

A technique for calculating the directional wave spectrum through fast Fourier transform (FFT) of an image and deriving wave information was developed [1]. Although this technique can derive spectra for wave period and wave direction information, it is difficult to identify the wave height value as the intensity of the radar image is used. To derive the wave height value, the signal-to-noise ratio (SNR) is calculated, and the wave height value is obtained through calibration with a buoy [4]. However, the wave height calculation using SNR is prone to errors when wind speed is low or outside the linear region due to first-order linear curve fitting [5, 6].

There has been a group of studies focused on estimating wave heights using traditional algorithms to overcome the limitations of the SNR-based method. [7] proposed a method utilizing empirical orthogonal functions instead of 3D FFT, achieving a correlation coefficient of 0.74 and a root mean square error (RMSE) of 0.52 m. [8] employed variational mode decomposition and attained a correlation coefficient of 0.87 and an RMSE of 0.38 m (without averaging).

There have been several studies utilizing neural networks to estimate wave heights with X-band radar images. [9] used a set of synthesized X-band radar images to train a convolutional neural network trained (CNN), achieving an RMSE of 0.39 m. [10] estimated significant wave heights using a multilayer perceptron with three features obtained from X-band radar images as input variables, achieving an RMSE of 0.22 m.

[11] proposed a model based on temporal convolutional networks (TCN). The authors achieved a correlation coefficient of 0.90 (without averaging), and an RMSE of 0.32 m by utilizing SNR and EEMD (ensemble empirical mode decomposition) features as inputs for the temporal convolutional networks.

A similar study was conducted by the same research group using a CNN and CGRU [12]. A CNN-based GoogleNet model with advanced ImageNet learning was introduced for pre-training, obtaining fine-tuning results suitable for wave measurement. Additionally, a convolution-gated recurrent unit (CGRU) was configured for time-series learning. This study proved that the prediction accuracy depends on the weather conditions. The correlation coefficients and RMSEs were 0.93 and 0.29 m, respectively when it had not rained, and 0.87 and 0.54 m when it rained. These improvements, however, had not been validated with large-sized datasets. [9] utilized the synthesized dataset, while [10–12] were validated with radar images of 9 days. [13] used RGB ocean images over 46 days, but the number of image sequences was limited to 3019.

There have been studies using satellite X-band SAR images that have similar characteristics to those of X-band radar images for ships. However, a significant difference exists in the presence or absence of shadowing areas due to differences in grazing angles. A method for deriving significant wave height based on a CNN using the corresponding images has also been studied [14]. Nonetheless, the resolution of SAR images is limited and may cause difficulties in interpreting waves within short periods.

In addition, research is being conducted on deriving significant wave heights based on a CNN algorithm using ocean wave surface images captured using a general-purpose camera [13]. Although an RGB camera has excellent resolution, light conditions at night and in poor weather are significantly different, limiting the ability to derive universal results.

The aim of this study is to introduce a method for directly inferring wave height through deep learning of radar images based on large datasets of buoy wave heights and radar images without applying the existing SNR in wave height calculations. In particular, a VGGNet-based CNN was used to directly learn the radar image, which has the advantage of being able to learn the temporal features of consecutive frames using a 3D CNN. Despite the simple network structure, this approach has the advantage of being able to learn spatiotemporal features together through a 3D CNN.

The study begins with a description of how and where the data was collected, followed by pre-processing, which includes a normalization process and data partitioning, and steps such as deep learning model training and inference. Finally, correlation coefficients, errors, etc. were analyzed and validated.

## Materials and method

### Data acquirement

**Fig 1:**
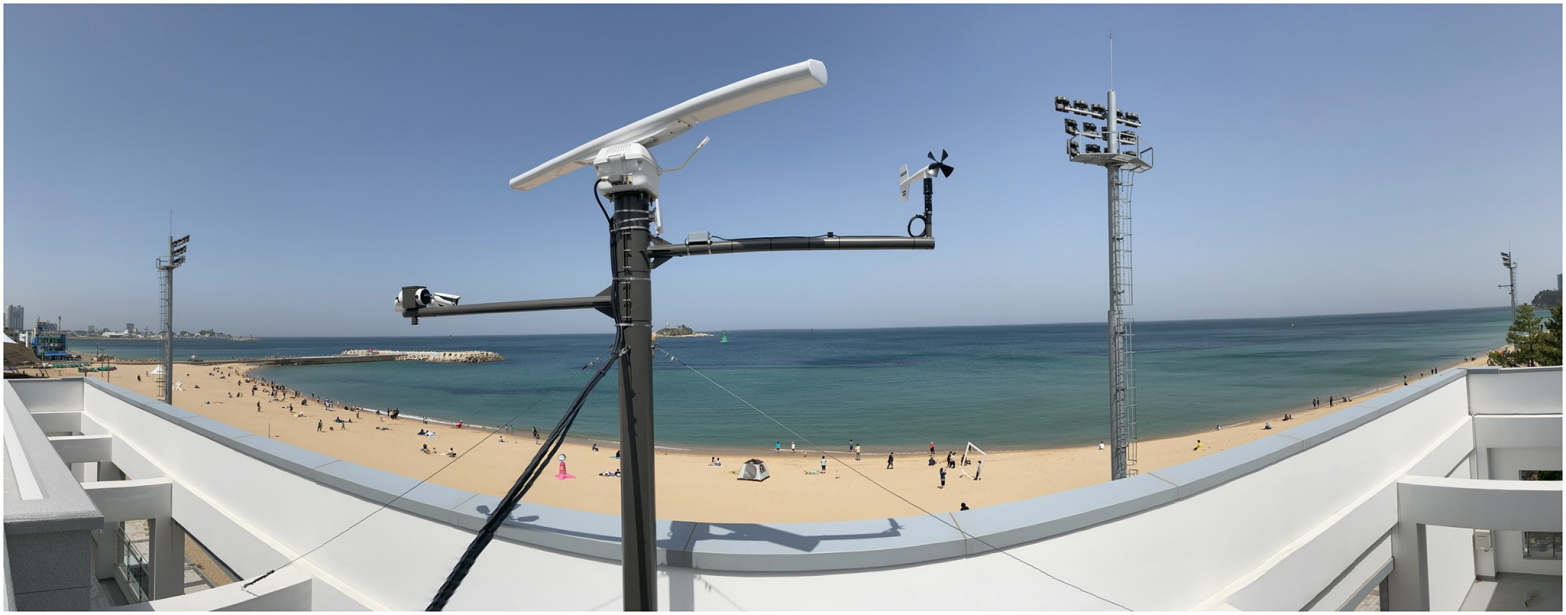
Radar site with X-band marine radar antenna and measurement areas.

In this study, radar images collected in Sokcho, Republic of Korea, were used as training data. Data were collected by installing a radar system on the roof of the Administrative Welfare Centre located at Sokcho beach, and the data used in this study were from June 1, 2021, to August 13, 2021 (Fig 1). X-band radar and an anemometer were used to collect and analyze the radar images. Data were collected through the X-band radar, and values such as wind direction and wind speed obtained through a wind sensor were used for radar analysis [5].

The X-band radar used in the study is the ARGUS-25X from SIMRAD. This magnetron-type radar has an output of 25kW, uses a 9ft long slot array antenna, and has HH polarization. The radar was set to short-pulse mode for measuring the sea surface. The radar outputs four types of signals in analog voltage—heading, trigger, bearing, and video. These signals are converted to A-scan radar images through an analog-to-digital converter installed on a PC. The radar images can be stored with a maximum resolution of 14 bits, sampled at 50MHz, and have angular and distance measurement capabilities within 1 degree and up to 3m. In this study, which focuses on significant wave height measurement, the images were down-sampled to 8 bits and 25MHz. Water depth varies in the coastal environment, with the radar analysis area depth ranging between 15 and 25 meters.

**Fig 2:**
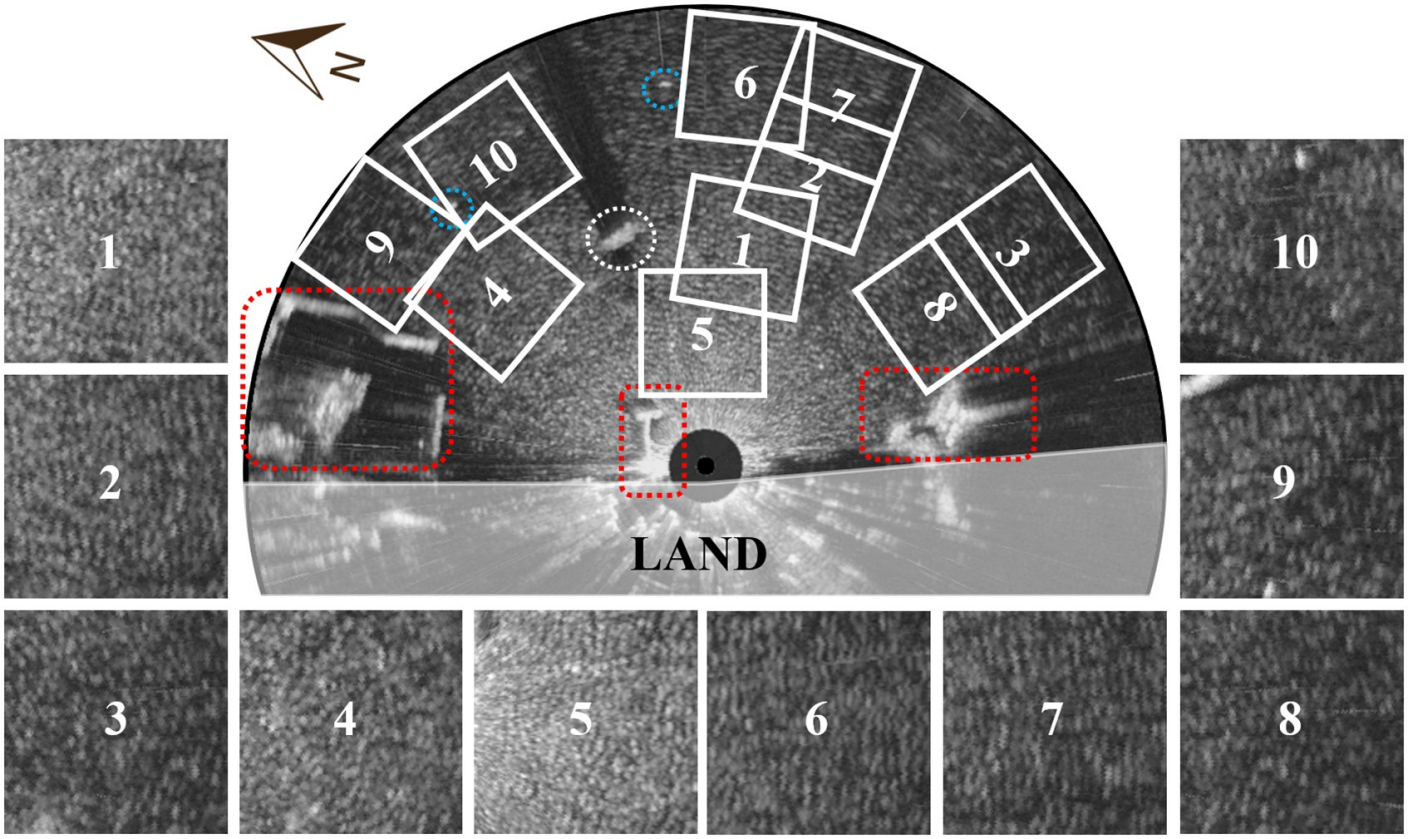
Example of radar image with analysis areas and obstacles.

An A-scan, which is a raw signal without filtering, is acquired in real-time through the devices mentioned above, with each radar image collected at approximately 1.43 s (42 rpm) intervals. The data collected is saved as an image file in the form shown in Fig 2. The 10 areas were set up by avoiding obstacles and points with abrupt depth changes based on the electronic nautical chart. In Fig 2, the blue dashed lines represent buoys, the white dashed lines represent islands, and the red dashed lines represent breakwaters and coastal areas. In the 10 sub-areas, the wave patterns can be observed separately.

**Fig 3:**
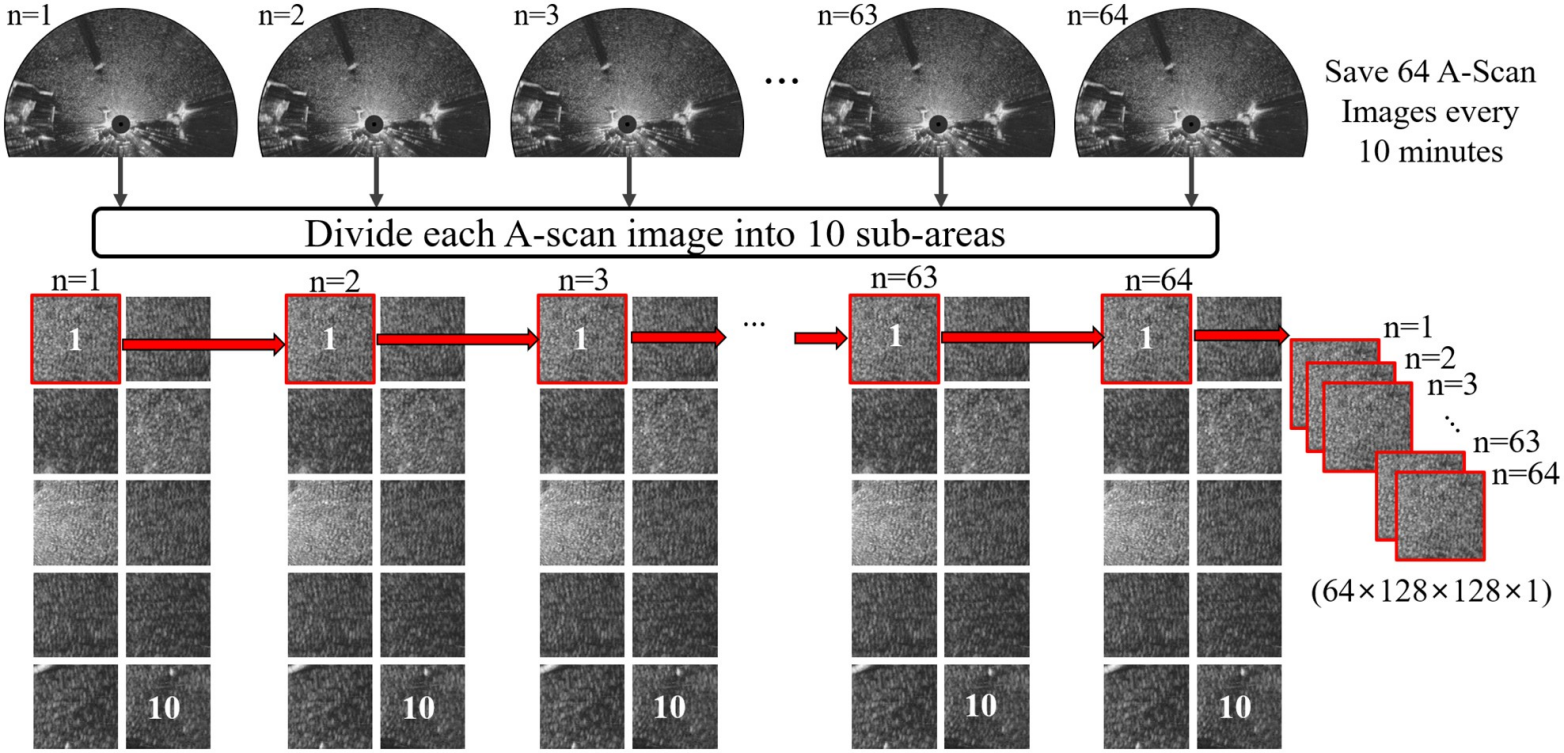
Process of radar image data collection.

**Fig 4:**
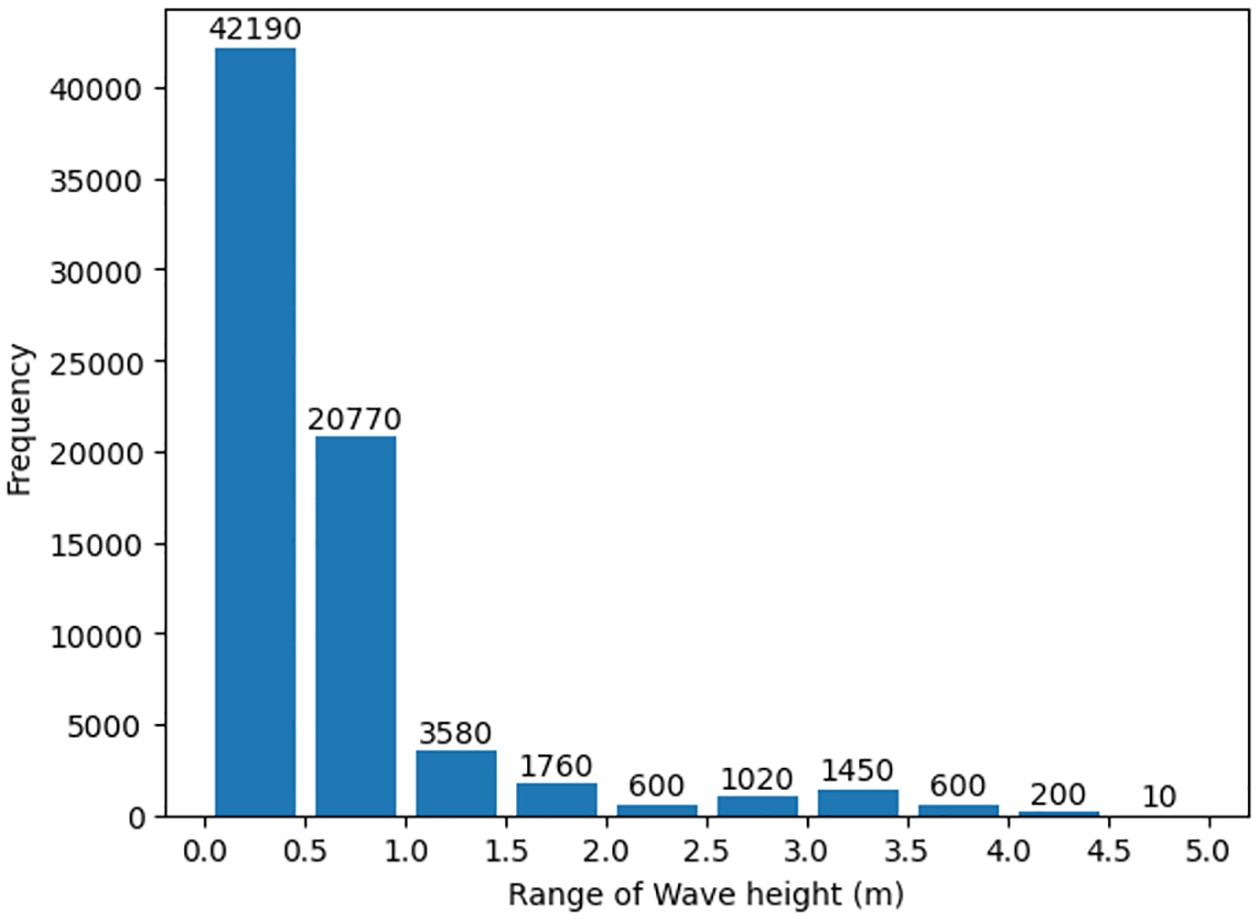
Distribution of data by significant wave height.

The following describes the data collection process and shows the overall process in Fig 3. The data used in this study consist of three-dimensional images of the same area (128 × 128), with 64 images per group placed in chronological order. Therefore, the input shape is (64, 128, 128, 1). Data for which no values were recorded were excluded, and as a result, 72,180 data were used in the study. Fig 4 shows the data distribution when dividing the entire significant wave height value corresponding to each piece of data into 10 random ranges. The number of data with the lowest significant wave height range was 43,670, accounting for more than half of the total number of data. The number of data with the highest significant wave height range was only 10, which is relatively small. Overall, the collected data were heavily distributed within the low significant wave height range, with very few data corresponding to the high significant wave height range. The data used in this study suffer from severe data imbalance.

### Target data acquirement

In this study, the wave height measured from an ocean buoy is used as the ground truth data. However, since the wave height measured at the buoy can change greatly within a short period of time, it is not appropriate to use this value directly as an answer for the data measured over 1-minute and 30-second periods. Therefore, this study uses the significant wave height as the ground truth value, which can better reflect the overall wave height characteristics. The significant wave height is defined as the first moment of the upper-third of the spectrum, obtained by collecting peak values of zero-crossing waves within a time series for a certain time [15]. To convert the range of significant wave heights corresponding to each data into values between zero and 1, we use Min-Max normalization. This technique adjusts the scale of the data and prevents the weights from being biased toward a specific feature as shown in (1) [16].

$$\begin{array}{c} x_{norm}=\frac{x-x_{min}}{x_{max}-x_{min}} \end{array}$$ (1)

### Data selection for network training

The dataset used in this study has a severe data imbalance in wave heights, with an overwhelming amount of data falling in the range of low significant waves. If used for training as is, overfitting to the more frequently occurring low wave height data would be highly likely. Therefore, to avoid this issue, we divided the entire dataset into three data groups (Level 1, 2, and 3) according to significant wave height. The significant wave height range for each data group is defined in Table 1. The numbers of instances of the levels 0, 1, and 2 were 67,540, 3,850, and 950, respectively.

**Table 1:** Range of significant wave height for each class.

class	Number of instances	significant wave height (m)	
		min	max
LEVEL 0	67,540	0	1.70
LEVEL 1	3,850	1.71	3.42
LEVEL 2	950	3.43	5.00

We separate the data for training, validation, and testing at each level. For each level, 190 data instances, which represent 20% of the number of Level 2 data with the smallest data rate, were randomly selected for validation and testing, respectively. Consequently, 570 data instances were used for validation, and another 570 data instances were used for testing. The remaining data were used for training.

To address the data imbalance, we randomly selected the same number of data (N) from each level of the training data to form data patches for each training iteration. The training data at each level were divided before selecting the data patches, ensuring that each divided group contains N data instances.

**Fig 5:**
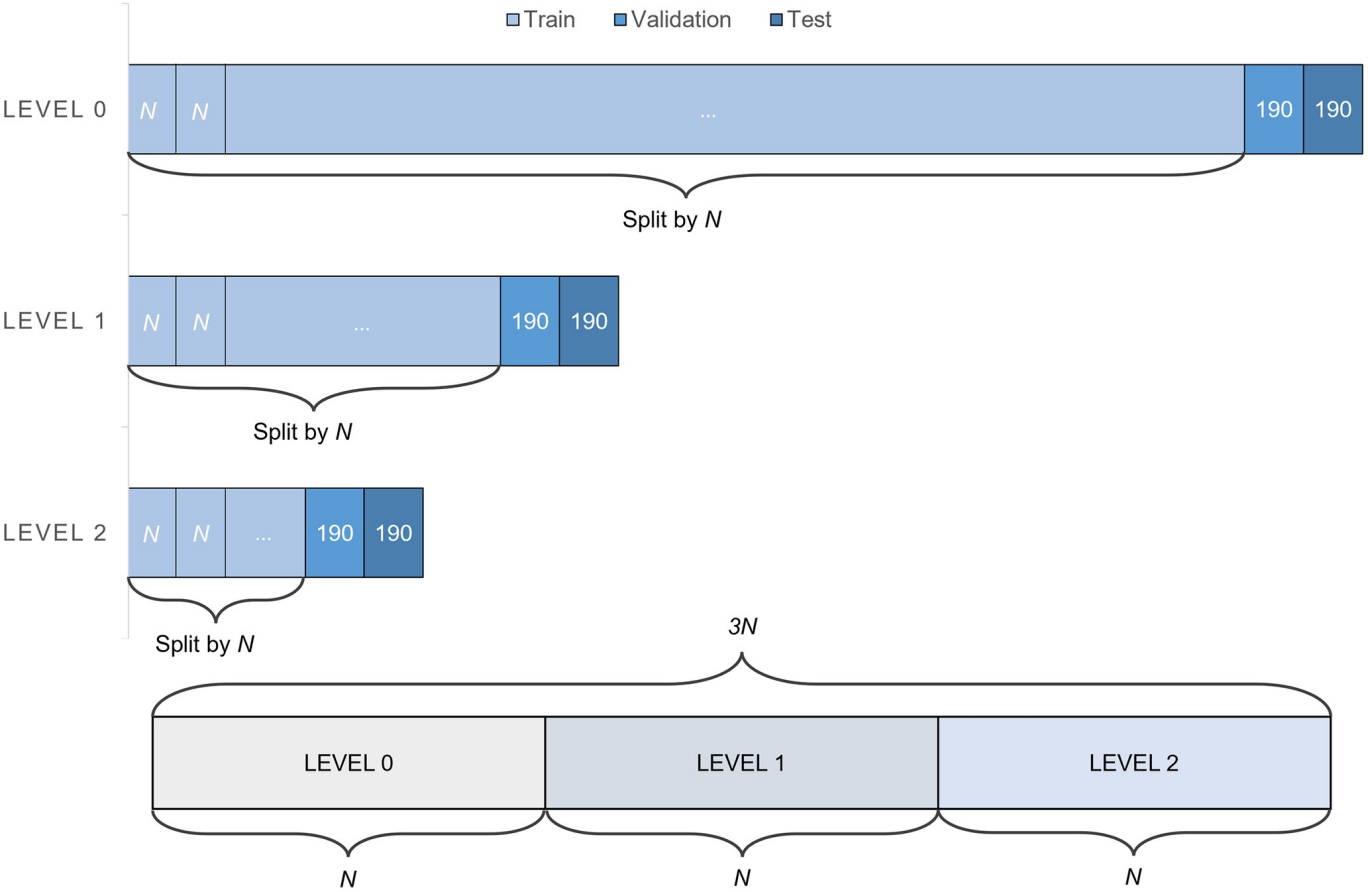
Data patch for training, (upper)data separation of training, validation, and test. Training data is separated into *N* segments, (lower)the composition of the data patches trained in one iteration.

This method resolves the data imbalance problem based on wave heights and prevents overfitting by enabling the learning of various data through iterations. Fig 5(upper) shows the data division process, and Fig 5(lower) shows the composition of the data patches trained in one iteration. To prevent data loss in the level with the smallest number of data, N below is set as a divisor of the number of data in the level 2.

### Network architecture

The aim of this study is to implement a deep learning regression model based on a CNN to predict significant wave height values for radar images. A CNN is an appropriate method for learning image data, as it can extract features while preserving the shape and spatial information of the input data [17].

**Fig 6:**
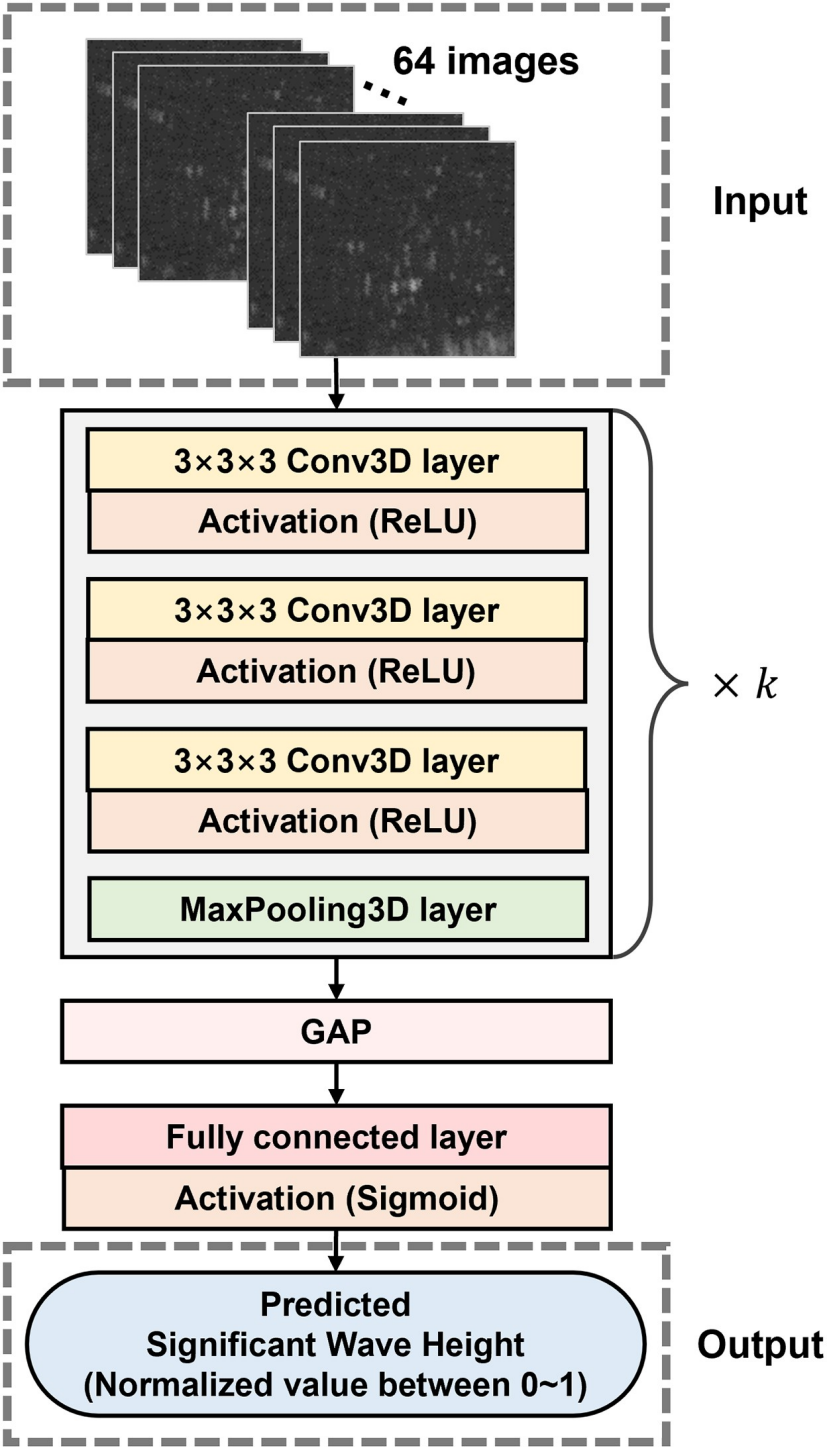
Deep learning network architecture for regression.

A CNN model was designed to extract features from a series of radar images Fig 6. Because each data instance has 64 images in sequence, we employed three-dimensional convolutions (Conv3D) to extract temporal features together with the special features. The model takes 64 images arranged in chronological order as input and interprets the features extracted using the Conv3D layer through a fully connected FC layer.

The proposed network model has a sequential structure of multiple convolutional blocks and one FC layer. It has a similar structure to VGGNet in the aspect of using convolution layers sequentially [18]. The model progressively learns complex features by repeatedly stacked convolutional blocks, which consist of multiple convolutional layers and one max pooling layer. The model utilizes 3D convolutional layers to effectively learn temporal features of image sequences and sigmoid function was used for the activation function of the output layer to perform the regression task. The loss function in the training process was the mean squared error (MSE). Max pooling layers of the proposed model reduce the number of training parameters and prevents overfitting by extracting more useful features for training [19]. The pool size was set to 2 to maximize the depth, which is the same as the VGGNet. Setting the pool size 2 and utilizing ReLU as the activation function is widely used for image recognition tasks to enhance accuracy [17, 20].

The network used in this study consists of *k* consecutive convolutional blocks and one FC layer. The features extracted by the convolutional layer are transformed into one-dimensional data by the global average pooling (GAP) layer, which is passed to the FC output layer. GAP converts the feature map into a one-dimensional vector, like the flatten layer but is known to improve the performance of the network compared to the flatten layer [21]. The FC output layer uses sigmoid as the activation function for the regression task, and the number of output nodes is one.

Because the output of the network is the normalized value of the significant wave height between 0 and 1, a post-processing of estimating the real value is necessary. The real value was estimated by the inverse equation of min-max normalization.

## Results

To evaluate the model’s performance, a comparison of results based on network architecture and learning parameters was conducted to identify the optimal structure and learning parameters. The model performance evaluation described in this study is based on the correlation coefficient between the predicted value for the test data and the actual significant wave height of the ground truth value. The correlation coefficient has a value between -1 and 1, with a value closer to 1 indicating greater accuracy [22].

Initially, performance is compared based on the value of *k*, which represents the number of convolutional blocks. During training, 1000 iterations were applied, with *N*, the number of dividing data, set at 10, and the batch size at 3 × *N*. Table 2 displays the hyper-parameters for each case.

**Table 2:** Hyper parameter of deep learning model for evaluating.

Number of CNN blocks (*k*)	Number of filters	Filter size	Strides	Padding	Pool size	Pool strides
*k* = 1	(64, 64, 128)	(3, 3, 3)	(2)	same	(2, 2, 2)	(2, 2, 2)
*k* = 2	(16, 32, 32, 64, 64, 128)	(3, 3, 3)	(2, 1)	same	(2, 2, 2)	(2, 2, 2)
*k* = 3	(8, 8, 16, 16, 32, 32, 64, 64, 128)	(3, 3, 3)	(2, 1, 1)	same	(2, 2, 2)	(2, 2, 2)

Here, *k* is greater than 1, and in this experiment, the study was conducted by dividing into three cases when *k* was 1, 2, and 3. Because the learning data was divided into constant values and randomly selected and used, the results varied slightly each time the experiment was repeated. To ensure unbiased results, 10 trials were performed for each case.

Table 3 shows the training outcomes based on the number of CNN blocks. As the random seed was fixed in each experiment, the data selection method in each trial was consistent. The experiment’s results indicate that the best performance was obtained with a correlation coefficient of 0.9647 and an average of 0.9540 when *k* = 2. When *k* = 3, better outcomes were achieved than when *k* = 1, but a slightly lower correlation coefficient was recorded with *k* = 2, possibly due to overfitting caused by the excessively deep network layer.

**Table 3:** Comparison of correlation coefficient according to numbers of CNN blocks.

	Trials										Best	Avg	Std
*n*	1	2	3	4	5	6	7	8	9	10			
*k* = 1	0.9008	0.9148	0.9118	0.9267	0.8899	0.8967	0.9018	0.9032	0.8672	0.9341	0.9341	0.9047	0.0179
*k* = 2	0.9543	0.9594	0.9556	0.9590	0.9598	0.9460	0.9498	0.9619	0.9299	0.9647	0.9647	0.9540	0.0096
*k* = 3	0.9534	0.9577	0.9559	0.9459	0.9470	0.9409	0.9362	0.9570	0.9515	0.9589	0.9589	0.9504	0.0073

The results based on *N*, the number of data groups utilized in a single iteration of data at a specific level, were compared next. The value of *N* was a factor of 570, the number of data used as training data among Level 2. Finding an appropriate value of *N* is crucial to avoid the risk of overfitting. The large value of *N* decreases the number of data in an iteration, and this may cause overfitting. The network architecture and hyper-parameters were the same as those listed in Table 2 for *k* = 2. Similarly, 10 experiments were conducted for each case, and the outcomes were compared.

Table 4 presents the training results for different values of *N*. As indicated in the table, the optimal results were achieved with *N* = 57, which had a correlation coefficient of 0.9697 and an average of 0.9639. We set the maximum number of *N* as 57 in the experiments due to the memory limitations during the training process.

**Table 4:** Comparison of correlation coefficient according to value of *N*.

	Trials										Best	Avg	Std
*n*	1	2	3	4	5	6	7	8	9	10			
5	0.9453	0.9561	0.9562	0.9457	0.9247	0.9367	0.9343	0.9494	0.9504	0.9544	0.9562	0.9453	0.0099
10	0.9543	0.9594	0.9556	0.9590	0.9598	0.9460	0.9498	0.9619	0.9299	0.9647	0.9647	0.9540	0.0096
15	0.9591	0.9596	0.9618	0.9627	0.9581	0.9495	0.9562	0.9578	0.9582	0.9662	0.9662	0.9589	0.0042
30	0.9659	0.9650	0.9663	0.9643	0.9617	0.9529	0.9576	0.9603	0.9636	0.9658	0.9663	0.9623	0.0041
57	0.9666	0.9646	0.9635	0.9695	0.9579	0.9506	0.9596	0.9669	0.9697	0.9697	0.9697	0.9639	0.0059

To examine the impact of the number of iterations on accuracy, we used the same network architecture and hyperparameters presented in Table 2 for *k* = 2 and *N* = 57. We conducted 10 experiments for each scenario and compared the results. Table 5 displays the training outcomes for different numbers of iterations. The best result was achieved with 2000 iterations, which had a correlation coefficient of 0.9699 and an average of 0.9642. As the number of iterations increased, the correlation coefficient tended to improve. However, when we set the number of iterations to 2500, the correlation coefficient decreased, suggesting that overfitting may have occurred.

**Table 5:** Comparison of correlation coefficient according to iteration.

	Trials										Best	Avg	Std
*n*	1	2	3	4	5	6	7	8	9	10			
500	0.9518	0.9584	0.9598	0.9568	0.9553	0.9458	0.9492	0.9602	0.9615	0.9641	0.9641	0.9563	0.0055
1000	0.9666	0.9646	0.9635	0.9695	0.9579	0.9506	0.9596	0.9669	0.9697	0.9697	0.9697	0.9639	0.0059
1500	0.9615	0.9697	0.9619	0.9695	0.9595	0.9526	0.9584	0.9678	0.9673	0.9695	0.9695	0.9637	0.0055
2000	0.9649	0.9675	0.9634	0.9692	0.9600	0.9582	0.9604	0.9620	0.9699	0.9668	0.9699	0.9642	0.0039
2500	0.9646	0.9664	0.9614	0.9677	0.9563	0.9613	0.9514	0.9614	0.9677	0.9656	0.9677	0.9624	0.0050

### Comparison with other methods

This section compares the results of significant wave height estimation of the proposed method to conventional models. As discussed in the introduction, utilizing SNR is one of the most popular methods to predict wave heights. It estimates the significant wave height by analyzing the signal-to-noise ratio obtained by the 3D FFT analysis of the radar images. A linear equation to regress the wave height is derived in the training phase, and the equation is validated with test data.

We utilized three popular deep learning models for the comparisons: ResNet50, InceptionV3, and InceptionResNetV2. These models have been utilized in recent studies for image recognition. These models were evaluated with or without pre-trained weights by the ImageNet dataset. The effects of 3D convolutions were validated by comparing the results to the model with 2D convolutional layers. All the structures of the model were the same as the proposed models except the dimensions of convolutions. For precise comparisons, all the hyperparameters of deep learning models were set to the same as the best case of the proposed method. The division of the training and test data was the same over all the methods.

Table 6 shows the correlation coefficients of the methods. The experiments were conducted 10 times, and the mean and standard deviations were listed in the table. As shown, the proposed model performed better than the other methods (*p* ≤ 0.01). It was confirmed that the proposed model was not significantly affected by random seeds by showing the most stable results. This indicates that the proposed model is able to learn the characteristics of radar image sequences accurately by avoiding the biases derived from specific data.

**Table 6:** Comparison of correlation coefficients according to iteration.

			Trials										Best	Avg	Std
Model		Weights	1	2	3	4	5	6	7	8	9	10			
SNR			0.7001	0.6851	0.6980	0.7051	0.6886	0.6864	0.6653	0.6689	0.6971	0.7021	0.7051	0.6653	0.0137
2D	ResNet50	ImageNet	0.9020	0.9341	0.9143	0.9302	0.9358	0.8851	0.9395	0.9253	0.9571	0.9255	0.9571	0.9249	0.0192
		None	0.9403	0.9396	0.9420	0.9150	0.9233	0.9284	0.9207	0.9134	0.9467	0.9235	0.9467	0.9293	0.0114
	InceptionV3	ImageNet	0.9630	0.9564	0.9608	0.9639	0.9599	0.9332	0.9516	0.9508	0.9546	0.9599	0.9639	0.9554	0.0085
		None	0.9398	0.9532	0.9516	0.9597	0.9424	0.9309	0.9346	0.9549	0.6397	0.9483	0.9597	0.9155	0.0924
	Inception ResNetV2	ImageNet	0.9648	0.9589	0.9612	0.9567	0.9448	0.9442	0.9376	0.9449	0.9574	0.9632	0.9648	0.9534	0.0091
		None	0.9422	0.9485	0.9539	0.9404	0.9307	0.9354	0.9346	0.9531	0.9422	0.9508	0.9539	0.9432	0.0077
	2D CNN		0.9560	0.9390	0.9563	0.9503	0.9441	0.9429	0.9376	0.9404	0.9021	0.9616	0.9616	0.9430	0.0157
ANN	Correlation Coefficient		0.9616	0.9642	0.9689	0.9683	0.9596	0.9669	0.9634	0.9619	0.9641	0.9679	0.9689	0.9647	0.0030
	RMSE		0.4181	0.4272	0.3940	0.4120	0.4331	0.4222	0.4673	0.4116	0.4236	0.4177	0.4673	0.4227	0.0180
	MAE		0.3057	0.3167	0.2899	0.3033	0.3059	0.3107	0.3462	0.3058	0.3179	0.3086	0.3462	0.3111	0.0138
	MSE		0.1748	0.1825	0.1552	0.1697	0.1876	0.1782	0.2184	0.1694	0.1794	0.1745	0.2184	0.1790	0.0155
3D	Correlation Coefficient		0.9649	0.9675	0.9634	0.9692	0.9600	0.9582	0.9604	0.9620	0.9699	0.9668	0.9699	0.9642	0.0039
	RMSE		0.3994	0.3858	0.4044	0.3527	0.4220	0.4389	0.4318	0.3907	0.3576	0.3840	0.4389	0.3967	0.0274
	MAE		0.2967	0.2907	0.2846	0.2683	0.3100	0.3114	0.3045	0.2875	0.2723	0.2837	0.3114	0.2910	0.0140
	MSE		0.1595	0.1489	0.1636	0.1244	0.1781	0.1926	0.1864	0.1526	0.1279	0.1474	0.1926	0.1581	0.0217

**Fig 7:**
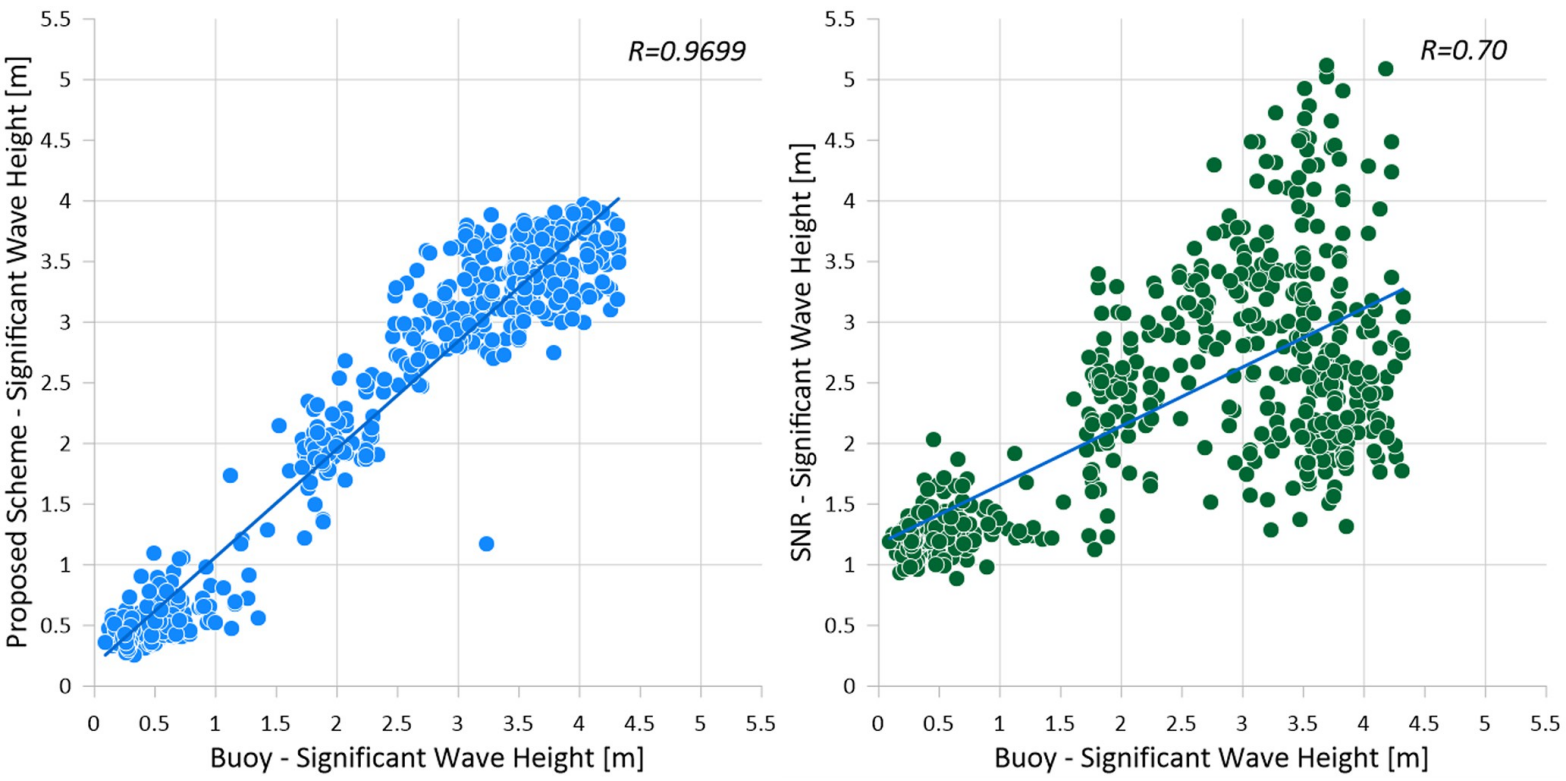
Comparison of significant wave height calculation between the proposed method (left) using the buoy data and the conventional method (SNR, right).

Table 6, among the proposed methods, the highest correlation coefficient (*R* = 0.9699) was depicted as shown in Fig 7(left). The X-axis represents the significant wave height of the buoy, which can be considered as the correct answer, while the Y-axis represents the significant wave height derived from the proposed method. The well-known SNR method for deriving significant wave height was also represented in the highest correlation coefficient result, as shown in Fig 7(right). For the SNR method, there is a tendency to overestimate wave height when the wind speed is low (3 *m*/*s* less) [23]. This is due to the backscatter that occurs from the sea surface [24].

Table 7 compares the state-of-the-art performances for significant wave height estimation using the maritime images in literature with the proposed method. It shows that the proposed method was validated with the largest dataset size recorded over 74 days, 20 times bigger than the conventional studies. Compared to conventional studies, the proposed method achieves the highest correlation coefficient (0.9699 for testing). The best coefficient among the conventional methods was 0.93 from [12], which was tested for the image taken when it had not rained. It should be noted that the higher coefficients of the proposed method were achieved with various weather conditions. The RMSE of the proposed method was relatively higher than in some previous studies. This means that the proposed method was able to learn trends of the wave heights, but the amount of estimation errors was relatively higher. This issue should be discussed with the characteristics of the datasets. The dataset was acquired from June to August, including the local rainy season. Consequently, it contains images under poor weather conditions, such as heavy rains or typhoons, which may decrease the model’s performance.

**Table 7:** Wave height estimation of the conventional method in literature.

Ref	Year	Method	Input data	Data collection periods	Number of Data	Correlation Coefficient	RMSE (m)	MAE (m)	MSE (m)
[9]	2020	2D CNN	Simulated X-band radar images	Simulated	-	-	0.39	-	-
[10]	2020	ANN	X-band radar images(SNR,Tp,wind condition)	9 days	576	0.89	0.22	-	-
[11]	2021	TCN	X-band radar image sequences	9 days	1448	0.90	0.32	-	-
[13]	2021	ConvLSTM	RGB images sequences	46 days	3019	-	0.1330	0.0886	0.0177
[12]	2022	CGRU	X-band radar image sequences	9 day	1448	0.93 (rainless) 0.87 (rainy)	0.29 (rainless) 0.54 (rainy)	-	-
[10]	2023	ANN	X-band radar images(SNR,Tp,wind condition)-our dataset	74 days	72180	0.9689	0.4227	0.3462	0.2184
Proposed	2023	3D CNN	X-band radar image sequences	74 days	72180	0.9699	0.4389	0.3114	0.1926

Additionally, to verify the reliability of the acquired data, the method introduced in [10] was used for interpretation. Specifically, the ANN method from [10] was applied to our radar data, and the results were summarized in the ANN section of Table 6 and the second row from the bottom of Table 7. In [10], SNR, peak wave period, and wind condition were used as input variables for constructing the ANN, and the activation functions for the hidden layer and output layer were set the same as in [10]. Using the method from [10], we achieved an average correlation coefficient of 0.9642, an average RMSE of 0.3967, an average MAE of 0.2910, and an average MSE of 0.1581.

In summary, when the proposed scheme and the method from [10] were evaluated using our dataset, their accuracy was found to be similar. However, the proposed scheme allows for results to be derived solely from radar image inputs, whereas the method from [10] requires additional pre-processing such as SNR, wave period, and wind information before results can be obtained through ANN.”

## Conclusion

Significant wave heights are important marine information in various fields that are directly or indirectly affected by the ocean because they are factors used in the design of ships, marine structures, coastal facilities, and so on. Buoys have been traditionally used for wave height measurement, but they require mooring and are expensive to install or operate in deep water areas. Estimating the wave heights using the signal-to-noise ratio of radar images is a popular technique in this field to substitute buoys, but the linearity of the method limits its accuracy in diverse situations in the ocean. There have been a few studies to estimate wave heights using deep neural networks recently, but the data sizes or the distributions of the radar images over the wave heights were limited.

In this study, we proposed a deep neural network model with 3D convolutions to estimate the significant wave height from X-band radar image sequences. The basic structure of the network was inspired by VGGNet and tuned to learn spatial characteristics of radar images, and 3D convolutions substitute 2D convolutions to capture the temporal features of the image sequences. Moreover, a technique of using a patch of data was introduced to train imbalanced data. In particular, the use of a large number of radar images over a period of about three months is a strength of this study compared to similar previous research.

Throughout this study, it was verified that the proposed method precisely estimates significant wave heights by achieving the mean correlation coefficients of 0.9642. It was significantly better than the conventional methods, whereas the SNR method showed coefficients of 0.6653. It was also confirmed that the proposed model achieved higher and more stable coefficients than popular network models or 2D convolutions significantly.

Using our data, we obtained results as good as those from the 3D CNN by applying the ANN introduced in [10]. However, our proposed method lets us immediately obtain significant wave height through 3D CNN from radar images. In contrast, using the ANN method requires additional pre-calculations such as the 3-dimensional FFT of radar images, SNR calculation through the dispersion relation, peak wave period calculation, and wind condition information.

In terms of the time required to calculate significant wave height, the conventional SNR method takes about 5 to 10 seconds, excluding the time it takes to read the radar image, as confirmed through direct participation in the development of a commercial system. This includes a three-dimensional Fast Fourier Transform, as it captures the energy characteristics. On the other hand, using the proposed method, it took 0.012 ± 0.001 seconds to derive the significant wave height (i9-12900K, GeForce RTX 3090, 100 repetitions), with the radar image reading time also excluded. This demonstrated a significant advantage in computation time, and it is judged that additional time investment for deep learning model training to improve calculation accuracy will be necessary.

This study focuses on deriving wave height from radar images using 3D CNN. Environmental factors affecting radar images include rain, snow, wind, among others. Further research is deemed necessary to understand the association of these factors and to improve the accuracy of wave height estimation.

One of the limitations of this study is that the actual wave heights were measured from a single buoy. The actual wave heights of the image spot could be slightly different according to the distance to the buoy. For more accurate wave estimation in the future, the significant wave estimation model should be implemented using only data from the area closest to the buoy. In our future study, we are planning to record radar images in longer periods and to utilize the images near the buoy to increase the reliability of the training data. Another limitation of the current study would be the requirement of memory. Because we utilized the three-dimensional convolutions, it would be difficult to adapt the model for embedded devices. Methods to lighten the proposed model could be studied as future work. It is expected that the proposed method could be used effectively for the controls of autonomous ships or various applications by overcoming these limitations.
